# Modelling the effect of short-course multidrug-resistant tuberculosis treatment in Karakalpakstan, Uzbekistan

**DOI:** 10.1186/s12916-016-0723-2

**Published:** 2016-11-18

**Authors:** James M. Trauer, Jay Achar, Nargiza Parpieva, Atadjan Khamraev, Justin T. Denholm, Dennis Falzon, Ernesto Jaramillo, Anita Mesic, Philipp du Cros, Emma S. McBryde

**Affiliations:** 1 School of Public Health and Preventive Medicine, Monash University, Melbourne, Australia; 2 Médecins sans Frontières, Manson Unit, London, UK; 3 National TB Institute, Ministry of Health, Tashkent, Uzbekistan; 4 Ministry of Health, Nukus, Uzbekistan; 5 The Victorian Tuberculosis Program at the Peter Doherty Institute, Melbourne, Australia; 6 Global TB Programme, World Health Organization, Geneva, Switzerland; 7 Médecins sans Frontières Holland, Amsterdam, The Netherlands; 8 James Cook University, Queensland, Australia

**Keywords:** Tuberculosis, Epidemiology, Treatment, Modelling, Multidrug-resistant tuberculosis, Extensively drug-resistant tuberculosis, Public health, Uzbekistan

## Abstract

**Background:**

Multidrug-resistant tuberculosis (MDR-TB) is a major threat to global TB control. MDR-TB treatment regimens typically have a high pill burden, last 20 months or more and often lead to unsatisfactory outcomes. A 9–11 month regimen with seven antibiotics has shown high success rates among selected MDR-TB patients in different settings and is conditionally recommended by the World Health Organization.

**Methods:**

We construct a transmission-dynamic model of TB to estimate the likely impact of a shorter MDR-TB regimen when applied in a low HIV prevalence region of Uzbekistan (Karakalpakstan) with high rates of drug resistance, good access to diagnostics and a well-established community-based MDR-TB treatment programme providing treatment to around 400 patients. The model incorporates acquisition of additional drug resistance and incorrect regimen assignment. It is calibrated to local epidemiology and used to compare the impact of shorter treatment against four alternative programmatic interventions.

**Results:**

Based on empirical outcomes among MDR-TB patients and assuming no improvement in treatment success rates, the shorter regimen reduced MDR-TB incidence from 15.2 to 9.7 cases per 100,000 population per year and MDR-TB mortality from 3.0 to 1.7 deaths per 100,000 per year, achieving comparable or greater gains than the alternative interventions. No significant increase in the burden of higher levels of resistance was predicted. Effects are probably conservative given that the regimen is likely to improve success rates.

**Conclusions:**

In addition to benefits to individual patients, we find that shorter MDR-TB treatment regimens also have the potential to reduce transmission of resistant strains. These findings are in the epidemiological setting of treatment availability being an important bottleneck due to high numbers of patients being eligible for treatment, and may differ in other contexts. The high proportion of MDR-TB with additional antibiotic resistance simulated was not exacerbated by programmatic responses and greater gains may be possible in contexts where the regimen is more widely applicable.

**Electronic supplementary material:**

The online version of this article (doi:10.1186/s12916-016-0723-2) contains supplementary material, which is available to authorized users.

## Background

Rifampicin-resistant tuberculosis and multidrug-resistant TB (MDR-TB; resistance to at least rifampicin and isoniazid) are among the greatest current threats to global TB control [1, 2]. An estimated 480,000 incident cases of MDR-TB occurred in 2014, but only 111,000 were reported to have been started on second-line treatment [3]. Globally, only half of those starting treatment complete it successfully, with many patients stopping treatment, not responding or dying. Therefore, about 10% of all incident MDR-TB cases globally are known to successfully navigate the complex pathway from presentation to detection and identification as multidrug-resistant, and subsequently through the difficult, toxic and costly treatment regimen.

The countries of Eastern Europe and the former Soviet Union report among the highest proportions of TB patients presenting with MDR-TB, both among new and retreatment cases [3]. In Uzbekistan, a former Soviet Union republic in Central Asia, TB prevalence was estimated at 122 (range, 61–204) per 100,000 population in 2014 [4]. About 7100 MDR-TB cases would be detectable among notified pulmonary TB cases, making it one of the 30 high-burden MDR-TB countries as defined by the World Health Organization (WHO). A national drug resistance survey conducted in 2011 found that 23% of new cases and 62% of previously treated cases had MDR-TB [5]. These levels varied within the country, and one region, Karakalpakstan in western Uzbekistan, with a population of 1.7 million, had the highest ratios (41% in new and 78% in retreatment cases). Since the early 2000s, Médecins sans Frontières (MSF) has been supporting the national TB programme of Karakalpakstan to strengthen TB surveillance, prevention and care. The model of care considers either inpatient or outpatient treatment, with a focus on providing early, supported ambulatory treatment, where possible.

Short-course regimens for MDR-TB (which typically consist of at least 9 months of fluoroquinolone, ethambutol, pyrazinamide and clofazimine, supplemented by high-dose isoniazid, kanamycin and prothionamide in the intensive phase) have been proposed and implemented in a number of settings in Africa and Asia [6–9]. Relapse-free treatment success rates of 84–90% and lower costs than currently recommended MDR-TB regimens have been reported in selected patient groups.

WHO has recently recommended the use of the shorter MDR-TB regimen only if patients do not have extrapulmonary TB, are not pregnant and if all medications from the shorter regimen are likely to be effective, based on the patient’s treatment history and the known or presumed resistance profile of the isolate [10, 11]. The efficacy of the regimen is currently being evaluated in a multicentre randomised controlled trial [12]. While uncertainties remain around the effectiveness of these regimens in some patient groups (e.g. children), they have stimulated much interest given the substantial boost they could provide to programmatic efforts if results obtained to date could be reproduced on a larger scale.

In 2013, MSF, in collaboration with the health authorities, commenced an observational study to measure the effectiveness of a shorter MDR-TB regimen in Karakalpakstan [13]. We present a mathematical model to estimate the likely impact of a 9–11 month MDR-TB regimen in Karakalpakstan on rates of disease and death, and compare this estimate with scenarios where alternative approaches are used to scale-up TB treatment programmes.

## Methods

**Fig. 1:**
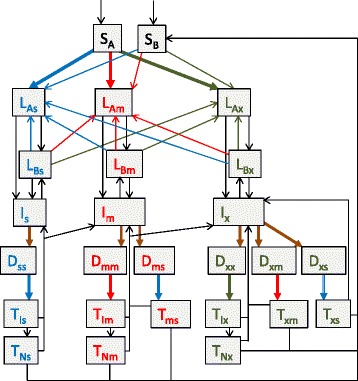
Model structure. Spontaneous recovery for patients in the detected compartments and all death flows are not depicted. Brown arrows represent case detection flows, the total of which are set equal for all strains. Hollow arrows represent treatment commencement flows, which are determined by the total number of persons awaiting treatment with that regimen and the availability of the regimen for each of the three regimens. Individual compartment names are explained in Additional file 1: Table S1 and summarised as follows: blue text and _s_ subscript, drug-susceptible TB; red text and _m_ subscript, multidrug-resistant TB; green text and _x_ subscript, XDR-TB (including also MDR-TB strains with resistance to fluoroquinolones or second-line injectable agents). S, susceptible to TB (_A_ and _B_ subscripts refer to fully susceptible and partially immune, respectively); L, latent infection (_A_ and _B_ subscripts refer to early and late latent infection, respectively); I, active TB disease in the community not yet detected; D, detected (first subscript refers to the actual resistance pattern of the infecting strain, second subscript refers to the strain thought to be present at diagnosis); T, on treatment (subscripts are as for D compartments for those incorrectly diagnosed, while for those correctly diagnosed _I_ subscript indicates still infectious on appropriate regimen, while _N_ subscript indicates no longer infectious). For simplicity, the model assumes no I_s_ patients are incorrectly detected as drug-resistant

The model structure is presented in Fig. 1 and the modelling approach is described in detail in Additional file 1, which lists compartment abbreivations (Additional file 1: Table S1) and parameter values (Additional file 1: Table S2). The model is based on our previous work [14] and incorporates a number of aspects that we consider important to modelling TB epidemiology in regions highly endemic for both TB and MDR-TB, including partial vaccine efficacy (leakiness) [15, 16], declining risk of active disease with time from infection, reinfection during latency, and acquisition of drug resistance through de novo amplification.

### Strains of TB modelled

Our existing model includes both MDR-TB and non-MDR-TB (henceforward DS-TB), with parameters for treatment duration and detection rates differing for each strain (note that a capital “S” indicates model compartments susceptible to infection, while a subscript “_s_” refers to antibiotic susceptibility.) While all rifampicin-resistant TB cases (including mono- and non-MDR-TB poly-resistant cases) are eligible for a full MDR-TB regimen [17], this analysis focuses on MDR-TB because rifampicin resistance is highly correlated with MDR-TB in the setting described [5] (note that the term “strain” does not necessarily refer to phylogenetically distinct lineages, but is used henceforward to refer to groups of *M. tuberculosis* organisms differing by drug resistance profile).

In order to consider the impact of programmatic approaches to improving MDR-TB control on the emergence of drug resistance, a third strain of TB is included within the model to represent patients ineligible for the short-course regimen. Henceforward, we use the abbreviation “XDR-TB” to refer to MDR-TB patients ineligible for the shorter regimen and “MDR-TB” to refer to patients with MDR-TB without additional resistance, although neither term accords directly with the corresponding microbiological definition. The inclusion of organisms with additional resistance beyond MDR-TB followed an approach analogous to that used to model MDR-TB by comparison to DS-TB, considering the acquisition of resistance as a progression (DS-TB → MDR-TB → XDR-TB). The model assumes that, although higher levels of resistance initially emerge through non-adherence to treatment and although a fitness cost is incurred by advancing resistance, all strains remain transmissible.

### Detection and treatment commencement

Separate compartments were used to distinguish the detection of cases of TB from the process of distinguishing the drug-susceptibility pattern of the infecting strain (Fig. 1). The first step in the diagnostic pathway consists of the patient’s presentation to the health system, which may be patient- or health system-related (e.g. due to false negatives in the diagnostic algorithm for the diagnosis of active TB). The model assumes that the rate of detection of persons with active TB is equal for all strains (moving from each I compartment to the corresponding linked D compartments), but that patients with resistant strains can then be misclassified with regards to their infecting strain according to the availability of diagnostics able to distinguish between MDR- and XDR-TB.

The proportion of individuals correctly identified with MDR-TB (D_mm_ ÷ [D_mm_ + D_ms_]) is determined by the availability and sensitivity of first-line drug resistance testing. This proportion is equal to the proportion of patients with XDR-TB who are diagnosed as having either MDR-TB or XDR-TB, as patients with XDR-TB are resistant to rifampicin and isoniazid by definition. Similarly, active XDR-TB correctly identified as MDR-TB patients may be correctly classified as XDR-TB depending on the availability of second-line drug resistance testing, or be incorrectly identified as MDR-TB (D_xm_) if only first-line drug resistance testing is available.

Patients awaiting treatment pass to the treatment compartments at a rate determined by the availability of the regimen they have been allocated. For example, for DS-TB regimens, this applies to all patients determined by the health service to have DS-TB (i.e. D_ss_, D_ms_ and D_xs_, who pass to T_Is_, T_ms_ and T_xs_, respectively). Patients appropriately commencing DS-TB regimens become non-infectious and ultimately recovered if retained on the regimen, with a proportion also dying and a proportion undergoing treatment interruption or failure (henceforward interruption/failure), returning to I_s_ or I_m_ depending on whether resistance amplification occurs. Similarly, patients awaiting appropriate MDR-TB and XDR-TB regimens transition from detected (D_mm_ and D_xx_) to infectious on treatment (T_Im_ and T_Ix_) to non-infectious (T_Nm_ and T_Nx_) as they progress through treatment.

The proportion of MDR-TB treatment interruption/failures resulting in resistance amplification to XDR-TB is assumed to be equal to that for DS-TB interruption/failures amplifying to MDR-TB. Patients whose strain has not been correctly identified and are commenced on an inappropriate treatment regimen have a low (but non-zero) treatment success rate and a modest reduction in infectiousness throughout the course of their treatment (Additional file 1: Table S2).

### Model calibration

In liaison with programmatic staff, the model was calibrated to the reported per capita TB incidence rate for Uzbekistan in 2015 [4], with secondary priorities, including historical consistency with TB burden in the region (particularly for more recent time points) and matching reported prevalence and mortality rates. MDR-TB was introduced into the model from 1977, such that it became a significant proportion of incident cases through the 1990s, consistent with its historical emergence. At the time of commencement of interventions in 2015, drug-resistant TB (MDR-TB or XDR-TB) constituted 23% of circulating strains [5], of which 29% were XDR-TB.

Next, the increasing availability of conventional MDR-TB treatment was simulated by scaling up the proportion of patients correctly identified as MDR-TB from 2005 to 2012. Treatment availability was capped at a maximum of 400 patients simultaneously on MDR-TB treatment regimens at any given point in time by 2012 (which became the predominant limiting factor around 2012), reflecting the current capacity of the program. This epidemiological calibration is presented visually in Additional file 1: Figure S1.

### Implementation of intervention and comparators

**Fig. 2:**
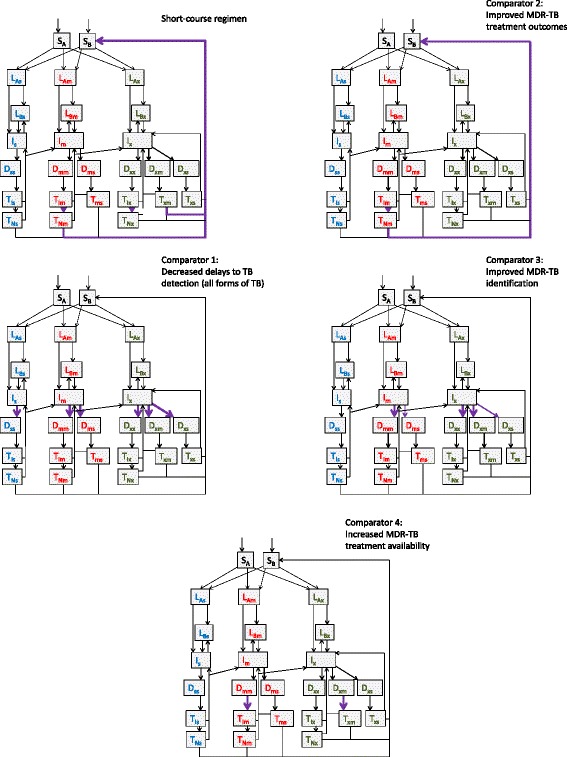
Implementation of main intervention and comparators. Model of the implementation of short-course MDR-TB and of the four comparator programmatic interventions. Increased flows highlighted by thick purple arrows, with indirect effects indicated through dashed purple arrows. For Scenario 4, the flows that are decreased are illustrated with thin purple arrows. Reinfection omitted

Table 1 presents the scenarios considered and Fig. 2 illustrates their implementation within the model. All intervention parameter values were increased sigmoidally from their baseline values in 2015 to reach their target values in 2017.

**Table 1:** Description of scenarios

Scenario	Programmatic implementation and evidence	Model implementation
1. Baseline programmatic conditions continued		All 2014 programmatic parameters remain unchanged (including 24 month duration of MDR-TB regimen and 400 treatment places available at any one time being the limiting factor for treatment commencement in 2014)
2A. Short-course MDR-TB regimen	Change from standard WHO regimen to short-course regimen [6–9]	Total period of time on treatment for MDR-TB regimens decreases from a mean of 24 months to 10 months (with treatment places remaining capped at 400)
2B. Short-course MDR-TB regimen with improved outcomes	As for short-course regimen, with improvement in treatment outcomes [6]	Treatment outcomes improve to a treatment success rate of 87.9% (with ratio of deaths to defaults under treatment unchanged), in addition to changes modelled under short-course regimen scenario above
3. Decreased delays to detection for all forms of TB (first comparator)	Active or intensified case finding halves the period of time to first presentation from baseline value [28, 29]	Time from disease onset to correct identification of patients as having active TB halves (with no change to the proportion correctly identified as to their infecting strain)
4. Improved MDR-TB treatment outcomes (second comparator)	Social support for all patients on treatment halves the proportion of outcomes resulting in interruption/failure or death [30]	Proportion of patients interrupting/failing or dying on treatment halves (with treatment success proportion increasing to 1 – [1 – previous treatment success proportion] ÷ 2)
5. Improved MDR-TB identification (third comparator)	Halve the number of health facilities without access to drug-susceptibility testing (e.g. Xpert MTB/RIF), thereby halving the proportion of patients not recognised as MDR-TB at presentation [31, 32]	Proportion of patients with MDR-TB who are incorrectly diagnosed as having DS-TB halves (with correct diagnosis proportion increasing to 1 – [1 – previous correct identification proportion] ÷ 2)
6. Increased MDR-TB treatment availability (fourth comparator)	Increased resources doubles the number of patients that can be simultaneously treated	Increase number of MDR-TB treatment places available to 800 (with DS-TB and XDR-TB treatment capacity unchanged)

*DS-TB* Drug-susceptible tuberculosis, *MDR-TB* Multidrug-resistant tuberculosis, *TB* Tuberculosis, *WHO* World Health Organization, *XDR-TB* Extensively drug-resistant tuberculosis

Short-course regimens for MDR-TB are implemented by decreasing the time spent in the MDR-TB treatment compartments (T_Im_, T_Nm_ and T_xm_) from 24 months to 10 months, as the short-course regimen can be completed in a minimum of 9 months. Treatment outcome proportions for both standard WHO and short-course regimens are assumed equal and parameterised to programmatic data on patient outcomes. As this is a highly conservative assumption given the improved treatment outcomes and maintenance of relapse-free survival often reported with the short-course regimen, simulations were repeated with an increase in treatment success rates to 87.9% [6].

Four comparator interventions were developed that modify other model parameters by a similar magnitude, are programmatically feasible and supported by evidence of efficacy. These scenarios are intended to put the magnitude of the response to the short-course regimen in context, rather than to definitively estimate the reduction in disease burden achievable by scaling up alternative programs.

### Outcomes

The main outcomes of interest resulting from the intervention and comparators are MDR-TB strain indicators (including absolute and proportionate incidence, prevalence and mortality).

### Sensitivity analyses

To better understand the effects of programmatic responses implemented simultaneously, we undertook a sensitivity analysis using Latin Hypercube Sampling to simultaneously vary the key parameters used in intervention implementation. Calibration remains unchanged, but the parameters used to simulate the alternative interventions from 2015 onwards are varied across plausible ranges divided into 10,000 equal sub-intervals.

An alternative set of analyses are presented to consider the programmatic impact of the same scenarios if the proportionate burden of MDR-TB has been underestimated, as could be inferred from the higher proportions of MDR-TB observed in Karakalpakstan in the 2011 drug resistance survey (although not statistically significantly different from the national estimate).

## Results

### Scenarios

**Fig. 3:**
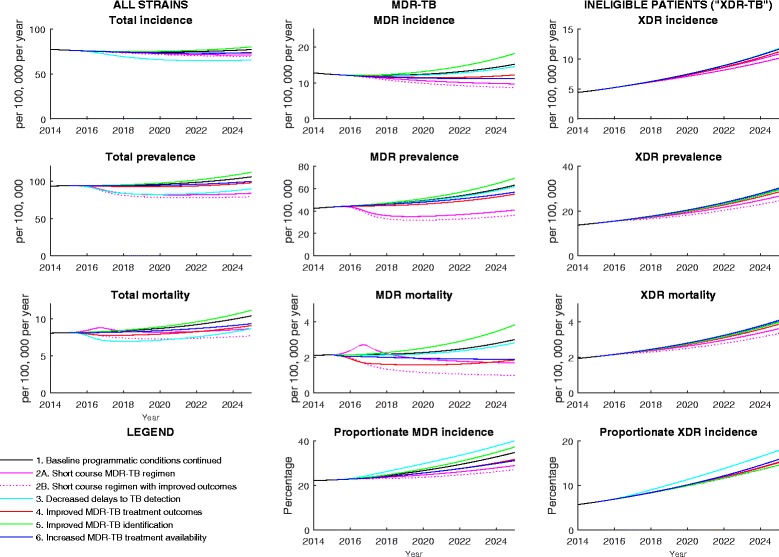
Scenario outcomes. Strains are presented by columns of panels and disease burden outcomes are presented by rows. Legend for all plots is presented in the lower left panel

Figure 3 and Table 2 present the results of the seven simulated Scenarios (baseline, two short-course MDR-TB regimen assumptions and four comparator interventions). Under the baseline Scenario, the resistant strains contribute an increasing proportion of disease over the 10 years to 2025, as their lower relative fitness is more than offset by their comparative advantages in diagnosis and treatment outcomes.

**Table 2:** Scenario results and percentage differences from baseline scenario in 2025

	1. Baseline	2A. Short-course regimen	2B. Short-course, improved outcomes	3. Decreased delays to detection	4. Improved MDR-TB treatment outcomes	5. Improved MDR-TB identification	6. Increased MDR-TB treatment availability
Total incidence^a^	77.2	71.1	69.4	65.6	73.9	80.2	73.3
% change		−8.0%	−10.2%	−15.0%	−4.4%	+3.8%	−5.1%
Total prevalence^b^	105.7	83.6	79.0	89.6	97.7	111.8	99.5
% change		−20.9%	−25.2%	−15.2%	−7.5%	+5.8%	−5.8%
Total mortality^a^	10.4	8.7	7.7	8.7	9.1	11.1	9.3
% change		−16.6%	−25.9%	−16.6%	−12.7%	+7.3%	−10.2%
MDR-TB incidence^a^	15.2	9.7	8.7	14.6	12.2	18.2	11.2
% change		−36.0%	−42.8%	−4.3%	−19.6%	+19.8%	−26.4%
MDR-TB prevalence^b^	63.0	40.8	36.1	61.6	55.0	69.2	56.7
% change		−35.2%	−42.6%	−2.2%	−12.7%	+9.9%	−9.9%
MDR-TB mortality^a^	3.0	1.7	1.0	2.8	1.9	3.8	1.9
% change		−43.9%	−67.2%	−6.2%	−38.2%	+27.5%	−37.1%
Proportion of incident cases MDR-TB	34.8	28.9	27.1	40.1	31.7	37.3	31.2
% change		−16.9%	−22.1%	+15.1%	−8.9%	+7.1%	−10.5%
XDR-TB incidence^a^	11.7	10.8	10.1	11.7	11.2	11.7	11.6
% change		−7.2%	−13.3%	+0.5%	−4.2%	−0.1%	−0.4%
XDR-TB prevalence^b^	29.8	26.6	24.5	30.0	28.5	29.3	30.2
% change		−10.8%	−17.7%	+0.5%	−4.4%	−1.6%	+1.2%
XDR-TB mortality^a^	4.0	3.6	3.3	4.1	3.9	4.0	4.1
% change		−10.5%	17.2%	+0.5%	−4.3%	−1.6%	+1.2%
Proportion of incident cases XDR-TB	15.1	15.2	14.6	17.9	15.1	14.6	15.9
% change		+0.8%	−3.5%	+18.2%	+0.1%	−3.8%	+5.0%

^a^Per 100,000 population per year

^b^Per 100,000 population

*MDR-TB* Multidrug-resistant tuberculosis, *XDR-TB* Extensively drug-resistant tuberculosis

Shortening the MDR-TB regimen duration has the greatest impact on MDR-TB burden of all interventions and has a significant effect on the overall TB burden in the region. A short-lived increase in TB and MDR-TB deaths is associated with the short-course regimen intervention. This results from a more rapid time to reaching the same outcomes (including death) than under the baseline conventional MDR-TB regimen scenario, and is not observed under the short-course with improved outcomes scenario. Under this scenario, some of the available treatment capacity (400 MDR-TB treatment places) is not filled due to faster throughput of patients.

Both decreased delays to TB detection and improved MDR-TB identification have no positive effect on MDR-TB indicators due to the absence of treatment availability for the increased number of identified patients under the programmatic conditions simulated. The impact of these two interventions on overall TB burden is also relatively small in this high MDR-TB burden setting. The greatest effect of improved MDR-TB treatment outcomes is on MDR-TB mortality, although its impact is small. Increased MDR-TB treatment availability results in improvements in MDR-TB burden broadly comparable to the change in regimen duration. None of the interventions simulated has a marked effect on absolute XDR-TB burden, with no significant increase in amplification from MDR-TB to XDR-TB observed through more rapid throughput of MDR-TB patients.

Additional file 1: Figure S2 illustrates the mechanisms of these interventions, indicating that doubling treatment places and decreasing regimen duration both result in treatment availability not being the limiting factor in patients starting treatment.

### Sensitivity and alternative analyses

The sensitivity analysis, which considers multiple programs implemented simultaneously, shows that several interventions have the potential to be synergistic (Additional file 1: Figures S4 and S5). For example, reducing MDR-TB misclassification can significantly reduce disease burden if combined with interventions that ensure that treatment is available to these patients once identified as MDR-TB. As decreasing time to presentation has a greater effect on DS-TB than MDR-TB, its effects on the absolute and relative burden of MDR-TB are opposite.

The alternative analysis under more pessimistic assumptions regarding the burden of MDR-TB (Additional file 1: Figure S3) further highlights the importance of increasing treatment availability or reducing regimen duration in improving MDR-TB burden in Karakalpakstan.

## Discussion

We find that implementing a 10-month treatment regimen for MDR-TB is among the most effective means for reducing the impact of this dangerous threat to global TB control in Karakalpakstan, a region with high rates of drug resistance among TB patients. The more rapid throughput of patients leads to an initial transient increase in mortality under the conservative assumption of unchanged treatment outcomes, although this is not a programmatically significant effect and is followed by a quick recovery and consistent decline in disease rates thereafter. Our model did not predict that wider use of the shorter MDR-TB regimen would increase the acquisition of additional drug resistance. The comparator intervention that led to reductions in MDR-TB disease burden most similar to expansion of the short-course regimen was doubling of MDR-TB treatment availability, while the other comparators were less effective. Moreover, synergistic effects could be expected if wider use of shorter MDR-TB regimens is combined with improved case detection.

Our first modelling study of TB transmission aimed to establish a flexible approach to simulating TB transmission dynamics in highly endemic settings within the framework of a deterministic compartmental model, but assumed regimen duration to be fixed for each strain. In this earlier work, we found that MDR-TB became the dominant strain at model equilibrium even in the presence of significant fitness costs, which is attributable to both lower rates of case detection and differences in progression through treatment [14]. In this study, we consider the issues surrounding the diagnostic process in greater detail, distinguishing detection of active TB from the process of determining the extent of drug resistance in the infecting organism and subsequent progress through the treatment regimen. The relative importance of each of these processes is likely to be setting-dependent and programs may act synergistically, as bottlenecks will exist at different points in the complex journey from active disease through to treatment completion.

Our conclusions depend on a number of model assumptions and the local TB epidemiology simulated. In particular, our modelling of a treatment program close to capacity explains the lack of effect observed from improved detection of TB cases and improved identification of MDR-TB patients from those detected. Additional file 1: Figure S2 shows that the reason for the relative ineffectiveness of most comparator interventions (all except increasing availability of MDR-TB treatment) is that they do nothing to relieve the bottleneck of treatment availability, such that numbers of patients awaiting treatment increase rapidly over the intervention period. Although there is no formal limit on MDR-TB treatment availability in Karakalpakstan, we consider expansion of the treatment program to manage the markedly increased patient load to be a programmatic intervention. As expected, doubling treatment availability and decreasing treatment time 2.4-fold had comparable effects on incidence and mortality, although the shorter treatment time had a greater effect on prevalence, as patients are considered prevalent cases until treatment is completed. The small increase in MDR-TB burden through improved detection is due to patients transitioning from being on inappropriate treatment for DS-TB (which is considered to have a partial therapeutic effect) to identified but awaiting treatment (and so untreated). Under- or over-estimation of the absolute or relative burden of each of the TB strains in the Province are likely to affect our conclusions, although only a markedly lower absolute burden of MDR-TB is likely to result in significant attenuation of the benefits from the shorter regimen. Given the complexity of the baseline dynamics simulated, we focused on programmatic parameters in our sensitivity analysis, rather than exploring variations of all parameters.

Our findings are likely to be generalisable to a number of other contexts in which treatment capacity is an important constraint, as the shorter regimen can be used in HIV-positive and paediatric populations. However, the programmatic situation is a key determinant of the regimen’s likely impact, as other factors may limit treatment commencement. For example, if MDR-TB treatment capacity is available but access to drug-susceptibility testing (DST) is limited and many patients are on incorrect regimens, improving access to DST is likely to compare more favourably to other interventions. Such situations may exist in contexts where intense community transmission of MDR-TB occurs, but DST is reserved for retreatment cases only. Alternatively, if extensive pre-health system delays to presentation are important in limiting the rate at which MDR-TB patients commence treatment, active case finding is likely to have a greater effect in reducing the burden of disease attributable to this strain. Therefore, in these situations, the shorter regimen may compare less favourably to these two interventions. Last, if poor treatment outcomes are reported programmatically, the shorter regimen may have a significant impact if improved treatment outcomes can be achieved, rather than by relieving the bottleneck to treatment commencement. Synergistic effects were observed in this study, which is understandable as both the shorter regimen and increased treatment capacity led to unfilled treatment capacity, which could be used if more patients with MDR-TB were detected by the health system and/or correctly classified as MDR-TB (Additional file 1: Figures S2, S4, S5).

A previous programmatic application of a similar model to Western Province of Papua New Guinea found a smaller impact of the short-course regimen [16]. However, in this earlier study, we considered treatment commencement to be dependent on the rate at which MDR-TB patients were detected, but independent of treatment availability. We also previously considered an extended period of hospitalisation to be necessary for implementation of the shorter regimen, due to the number of drugs employed during the intensive phase of treatment. Although this consideration is not explicitly modelled here, the community-based approach to treatment currently employed in Karakalpakstan would make scale-up of treatment (e.g. Scenario 6) less resource-intensive and more feasible than in settings where hospitalisation is deemed essential throughout the intensive phase.

We do not present an economic analysis and the comparator interventions are not intended to be equivalent in terms of resource consumption or expense. However, several may be considerably more difficult to implement and many of the resources already in place to provide the standard WHO regimen could be adapted to short-course treatment. In fact, the short-course intervention is likely to be significantly cost-saving, as we estimate the expense of the short-course regimen at around 760 Euros in Karakalpakstan by comparison to over 3000 Euros for the standard regimen (personal communication MSF), which is consistent with estimates from elsewhere [10, 18]. Therefore, even under scenarios that achieve a higher throughput of patients as a result of faster treatment completion, the short-course intervention should be cost saving due to its lower cost per unit time on treatment. By contrast, for programs such as active case finding, improved MDR-TB identification, patient monitoring for response to treatment and support for adverse effects, and increased treatment availability, significant additional resources are likely to be required.

The short-course regimen we consider is based on analysis of sequential cohorts of patients enrolled into treatment in Bangladesh and elsewhere [6–9]. The study and subsequent follow-up has demonstrated favourable outcomes sustained after treatment completion without significant amplification of resistance [9]. There has been debate over whether a regimen based on this form of evidence, rather than the gold standard of the randomised controlled trial, should be accepted for programmatic use. Therefore, a multi-centre, non-inferiority randomised controlled trial has been initiated to better determine the efficacy of safety of the regimen [12]. Such evidence will be of great use in determining the extent and speed with which such regimens should be adopted, particularly given that the standard regimens are based on very low quality evidence [19] and that meta-analyses of standardised regimens estimate treatment success rates around 50% (when including patients ineligible for shorter regimens) [20].

The recent WHO guidelines provide a conditional recommendation supporting the use of the shorter regimen in the context of further research, although the broader epidemiological impact of the regimen has not yet been observed. Modelling the likely effect of such a programmatic change is important in this context. This study aims to realistically simulate the introduction of short-course regimens for a similar patient group to that recommended for treatment by the WHO guidelines [10].

Local patterns of drug resistance are also an important consideration, as it is important to limit treatment to patients infected with strains susceptible to the constituents of the regimen as much as possible, to avoid further exacerbating drug resistance problems. Although evidence for the effectiveness of short-course MDR-TB regimens is now emerging from a range of settings [7], our study is not intended to determine the regimen’s efficacy, but rather to estimate likely improvements in MDR-TB control through shortening treatment duration.

Although new agents are now available for the treatment of MDR-TB [21, 22], these are largely intended to strengthen conventional MDR-TB regimens [23]. More important than the development of single agents is the formulation of new regimens to reduce treatment duration at the programmatic level. Therefore, trials of new shorter regimens, such as STAND (Shortening Treatment by Advancing Novel Drugs, NCT02342886), Practecal and Nix-TB, hold promise for improvements in TB treatment effectiveness [24–26]. Moreover, STREAM II (The Evaluation of a Standard Treatment Regimen of Anti-Tuberculosis Drugs for Patients with Multidrug-Resistant Tuberculosis, ISRCTN18148631), which considers several short-course regimens, includes a treatment arm in which injectable agents are avoided entirely [27]. Although proving the efficacy of these regimens is essential, it is also important to demonstrate that programmatic benefits are achievable to argue for their introduction.

## Conclusions

We find that short-course regimens hold substantial promise in reducing the overall burden of disease and death due to MDR-TB in Karakalpakstan and have the potential to be a major weapon in the fight against this strain. The context in which the regimen is introduced is a key determinant of its likely impact and changing to the shorter regimen is likely to be most beneficial in settings where treatment capacity is an important programmatic consideration. Implementation of the shorter regimen did not lead to a significant increase in the prevalence of more resistant strains (e.g. XDR-TB), although such strains limited the extent to which the shorter regimen could be applied.

## Additional files

Additional file 1: Full methods, including compartment abbreviations and parameter values. Additional figures to illustrate calibration approach, mechanisms of interventions' impact and sensitivity analysis. (DOCX 2488 kb)
Additional file 2: Model code for Matlab 2015b. (ZIP 10 kb)

## Abbreviations

DS-TB: Drug-susceptible tuberculosis
MDR-TB: Multidrug-resistant tuberculosis
MSF: Médecins sans Frontières
TB: Tuberculosis
WHO: World Health Organization
XDR-TB: Extensively drug-resistant tuberculosis Model compartment abbreviations are presented in Additional file 1: Table S2.
