# Supplementary material for: Modelling the effect of short-course multidrug-resistant tuberculosis treatment in Karakalpakstan, Uzbekistan
Source: BMC Med. 2016 Nov 18;14:187. doi: 10.1186/s12916-016-0723-2 (PMC5114735; doi:10.1186/s12916-016-0723-2)
Supplement: Additional file 1: — Full methods, including compartment abbreviations and parameter values. Additional figures to illustrate calibration approach, mechanisms of interventions' impact and sensitivity analysis. (DOCX 2488 kb) [file 12916_2016_723_MOESM1_ESM.docx]

# Online Supplement

Full Methods

Figure 1 (main text) presents the model structure and Table S1 lists the meaning of the compartment abbreviations.

Previous Model

The model is based on our previous work and incorporates a number of aspects that we consider important to modelling TB epidemiology in regions with high overall burden and a significant proportion of disease attributable to MDR-TB.[^1^](#_ENREF_1)^,^[^2^](#_ENREF_2) This base model consisted of ten compartments representing progression from susceptible (either fully susceptible to TB or partially immune) through two sequential latency compartments to active disease in the community. (Note that the term “susceptible” and compartments represented by a capital S refer to susceptibility to infection with TB regardless of its antibiogramme, whereas “DS-TB” and compartment labels with subscripts _s_, _m_, and _x_ refer to the drug-susceptibility of the infecting strain [respectively drug-susceptible TB, MDR-TB and XDR-TB; see Figure 1 {main text} and Table S1 for more detail]). From here, patients may die, spontaneously recover and remain at risk of disease, or be detected by the health system and commenced on treatment. Treatment may result in death, treatment interruption or failure with return to active disease, or completion of the regimen with return to a partially immune state. Model features retained from our previous work include: partial vaccine efficacy (leakiness),[^3^](#_ENREF_3) declining risk of active disease with time from infection, reinfection during latency and acquisition of drug resistance through *de novo* amplification.

Strains of TB Modelled

Our previous model included both MDR-TB and non-MDR-TB, with parameters for treatment duration and detection rates differing for each “strain”. (These are not necessarily strains of the organism in the phylogenetic sense. However, forms of TB exhibiting specific drug-resistance patterns are henceforward referred to as “strains”.) While all rifampicin-resistant TB cases (including mono- and non-MDR-TB polyresistant cases) are eligible for a full MDR-TB regimen,[^4^](#_ENREF_4) this analysis focuses on MDR-TB because rifampicin resistance is highly correlated with MDR-TB in the setting described.[^5^](#_ENREF_5)

In order to consider the impact of programmatic approaches to improving MDR-TB control on the emergence of drug-resistance, a third strain of TB is included within the model, to represent patients ineligible for the short-course regimen. This group is composed of patients who had previously received at least one month of second-line drugs and patients with additional resistance to fluoroquinolones or injectable agents or both (extensive drug resistance or XDR-TB). (For simplicity, in this work, we use the abbreviation “XDR-TB” to refer to MDR-TB patients ineligible for the shorter MDR-TB regimen, while the abbreviation “MDR-TB” is used to refer to patients with MDR-TB without additional resistance. Note that neither group accords directly with accepted microbiological definitions.) The inclusion of organisms with additional resistance beyond MDR-TB followed an approach analogous to that used to model MDR-TB by comparison to DS-TB, considering the acquisition of resistance as a progression (drug-susceptible → MDR-TB → MDR-TB with additional resistance and XDR-TB). The model assumes that although higher levels of resistance initially emerge through non-adherence to treatment and although a fitness cost is incurred by advancing resistance, all strains remain transmissible. Compartments representing any form of TB infection are thus replicated threefold: one for persons infected with strains which are not MDR-TB (denoted _s_); one for cases infected with MDR-TB (denoted _m_); and one for cases infected with MDR-TB strains with additional resistance to fluoroquinolones, second line injectable agents or both (denoted _x_).

Detection and Treatment Commencement

Separate compartments were used to distinguish the process required to identify a case of TB from the process of distinguishing the drug-susceptibility pattern of the infecting strain (Figure 1 [main text]). The first step in the diagnostic pathway consists of the patient’s presentation to the health system. Delays in this process can be patient- or health system-related (e.g. due to false negatives in the diagnostic algorithm for the diagnosis of active TB). In Karakalpakstan, the diagnostic algorithm for TB and drug-resistant TB is usually a combination of clinical judgement, sputum smear microscopy and liquid-culture based first and second-line drug-susceptibility testing. Detection of RR-TB has recently been reinforced by the broad scale-up of access to molecular diagnostics, largely Xpert MTB/RIF.[^6^](#_ENREF_6) The model assumes that the rate of detection of persons with active disease (I compartments) is equal for all strains of TB, such that the rate at which DS-TB patients are detected (moving from I_s_ to D_ss_) is equal to the rate that patients are correctly or incorrectly detected with MDR-TB (moving from I_m_ to either D_mm_ or D_ms_) and to the rate that patients with XDR-TB are correctly or incorrectly identified (moving from I_x_ to either D_xx_, D_xm_ or D_xs_).

Next, patients enter a detected (D) compartment that is determined both by their infecting strain and whether this strain was correctly identified. All detected DS-TB patients are assigned to a compartment of correctly diagnosed (D_ss_), considering that mis-diagnosis of DS-TB as MDR-TB is uncommon and that treatment outcomes for such patients are likely to be comparable to those for appropriately treated DS-TB. Active MDR-TB patients (I_m_), however, may either enter a compartment correctly diagnosed as MDR-TB (D_mm_) or incorrectly diagnosed as DS-TB (D_ms_). The proportion of individuals correctly identified with MDR-TB (D_mm_ ÷ [D_mm_ + D_ms_]) is determined by the availability and sensitivity of drug resistance testing able to identify MDR-TB (e.g. Xpert MTB/RIF, line probe assays and conventional drug resistance testing). This proportion is equal to the proportion of patients with XDR-TB who are diagnosed as either MDR-TB or XDR-TB, as patients with XDR-TB are resistant to rifampicin and isoniazid by definition. Active XDR-TB patients (I_x_) who have been correctly identified as MDR-TB patients may be correctly identified as XDR-TB if second line resistance testing is available (e.g. line probe assays and conventional drug resistance testing, entering D_xx_) or be incorrectly identified as MDR-TB (D_xm_) if only first line drug resistance testing is available.

Patients awaiting treatment pass to the treatment compartments at a rate determined by the availability of the regimen they have been allocated. For DS-TB regimens, this applies to all patients determined by the health service to have DS-TB (i.e. D_ss_, D_ms_ and D_xs_, who pass to T_Is_, T_ms_ and T_xs_ respectively). Patients appropriately commencing DS-TB regimens (T_Is_) become non-infectious (T_Ns_) and ultimately recovered (S_B_) if retained on the regimen, with a proportion also dying and a proportion undergoing treatment interruption or failure (henceforward interruption/failure, returning to I_s_ or I_m_ depending on whether resistance amplification occurs). Patients on an inappropriate DS-TB regimen (D_ms_) spend six months on treatment with a low treatment success rate, with most returning to active disease (I_m_). Similarly, patients awaiting appropriate MDR-TB and XDR-TB regimens transition from detected (D_mm_ and D_xx_) to infectious on treatment (T_Im_ and T_Ix_) to non-infectious (T_Nm_ and T_Nx_) as regimens become available.

Model Calibration

Death rates are set to the reciprocal of the life expectancy for Uzbekistan, with the crude birth rate then adjusted to match the population growth rate for the country. The initial starting population is adjusted so that the population reaches 1.7 million in 2015.

In liaison with programmatic staff, the model was calibrated to the reported overall TB incidence rate for Uzbekistan in 2015,[^7^](#_ENREF_7) with secondary priorities including historical consistency with TB burden in the region (particularly for more recent time points) and matching reported prevalence and mortality rates. First, the transmission parameter is adjusted to reach an incidence rate of 80 per 100,000 per year as treatment availability increases over the latter part of the twentieth century. Although MDR-TB is likely to have a reduced relative fitness compared to DS-TB, the magnitude of this cost is uncertain.[^8^](#_ENREF_8) Consistent with past research, a moderate fitness cost (relative fitness of 0.7) is conferred to this strain to prevent it rapidly becoming the predominant strain within a few years of its emergence. MDR-TB is introduced into the model from 1977, such that it becomes a significant proportion of incident cases through the 1990s (around 5% in 1990 to 10% in 2000), consistent with its historical emergence. At the time of the commencement of interventions in 2015, MDR-TB constitutes 23% of all circulating strains,[^5^](#_ENREF_5) of which 71% are eligible for the short-course regimen.

Next, the increasing availability of conventional MDR-TB treatment is simulated by scaling up the proportion of patients correctly identified as MDR-TB from 2005 to 2012, with treatment capped at the expected saturation limit of 400 patients simultaneously on treatment by 2012 (such that availability of treatment, rather than correct identification of MDR-TB, becomes the predominant limiting factor around 2012). Similarly, XDR-TB enters the model from 2001 onwards with treatment capped at 40 persons simultaneously on treatment by 2012. This epidemiological calibration is presented visually in Figure S1.

Implementation of Intervention and Comparators

Table 1 (main text) presents the Scenarios considered and Figure 2 (main text) illustrates their implementation within the model. All intervention-related parameter values are increased sigmoidally from their baseline values in 2015 to reach their target values in 2017.

Short-course regimens for MDR-TB are implemented through decreasing the time spent in the MDR-TB treatment compartments (T_Im_, T_Nm_ and T_xm_) from 24 months to ten months, as the short-course regimen can be completed in a minimum of nine months. Treatment outcome proportions for both standard WHO and short-course regimens are assumed equal and parameterised to programmatic data on patient outcomes. As this is a highly conservative assumption given the improved treatment outcomes and maintenance of relapse-free survival often reported with the short-course regimen, simulations were repeated with an increase in treatment success rates to 87.9%.[^9^](#_ENREF_9)

Four comparator (Scenarios 3 to 6) interventions were developed that modify other model parameters by a similar magnitude, are programmatically feasible and supported by evidence of efficacy. Scenario 3 consists of halving the total duration of delays to presentation and diagnosis for all forms of TB (i.e. doubling the total rate of transition from each I compartment to all the linked D compartments, without modifying the proportion of patients with resistant strains correctly identified). Scenario 4 consists of halving adverse MDR-TB treatment outcomes (i.e. interruption/failure and death) through more effective programmatic implementation of conventional MDR-TB regimens. Scenario 5 consists of halving the proportion of health facilities without access to first-line drug susceptibility testing (e.g. Xpert MTB/RIF), thereby increasing the proportion of patients correctly identified by infecting strain (D_mm_, D_xx_ and D_xm_) and decreasing the proportion incorrectly classified (D_ms_ and D_xs_). Scenario 6 consists of doubling the availability of MDR-TB treatment places from 400 to 800.

Outcomes

The main outcomes of interest resulting from the intervention and comparators are MDR-TB strain indicators (including absolute and proportional incidence, prevalence and mortality). Equivalent indicators for DS-TB and XDR-TB are also reported, to illustrate the impact of MDR-TB-focused action on other strains and the TB epidemic as a whole.

Sensitivity Analyses

To better understand the effects of programmatic responses if implemented simultaneously, we undertook a sensitivity analysis using Latin Hypercube Sampling to simultaneously vary the key parameters used in intervention implementation. Calibration remains as described above, but the parameters used to simulate the alternative interventions from 2015 onwards are varied across their plausible ranges divided into 10,000 equal sub-intervals.

An alternative set of analyses are presented to consider the programmatic impact of the same scenarios if the proportionate burden of MDR-TB has been underestimated, as could be inferred from the higher proportions of MDR-TB observed in Karakalpakstan in the 2011 drug resistance survey (although this was not statistically significantly different from the national estimate). To allow MDR-TB to constitute a greater proportion of resistant strains at the commencement of interventions, this strain is introduced to the model ten years earlier without modifying its relative fitness, such that MDR-TB constitutes around 46% of incident cases in 2011.

**Table S1 Compartment symbols and descriptions**

| **Symbol** | **Description** |
| --- | --- |
| S_A_ | Uninfected, unvaccinated, fully susceptible |
| S_B_ | Uninfected, vaccinated or previously treated, partially susceptible |
| L_As_ | Early latency, DS-TB |
| L_Bs_ | Late latency, DS-TB |
| I_s_ | Active infection, undetected, DS-TB |
| D_ss_ | Active infection, detected, awaiting treatment, DS-TB |
| T_Is_ | On treatment, still infectious, DS-TB |
| T_Ns_ | On treatment, no longer infectious, DS-TB |
| L_Am_ | Early latency, MDR-TB |
| L_Bm_ | Late latency, MDR-TB |
| I_m_ | Active MDR-TB, undetected |
| D_mm_ | Active MDR-TB, detected and awaiting correct treatment |
| T_Im_ | On treatment, still infectious, MDR-TB |
| T_Nm_ | On treatment, no longer infectious, MDR-TB |
| D_ms_ | Active MDR-TB, incorrectly identified as DS-TB |
| T_ms_ | Active MDR-TB on inappropriate DS-TB regimen |
| L_Ax_ | Early latency, XDR-TB |
| L_Bx_ | Late latency, XDR-TB |
| I_x_ | Active XDR-TB, undetected |
| D_xx_ | Active XDR-TB, detected and awaiting correct treatment |
| T_Ix_ | On treatment, still infectious, XDR-TB |
| T_Nx_ | On treatment, no longer infectious, XDR-TB |
| D_xm_ | Active XDR-TB, incorrectly identified as MDR-TB |
| D_xs_ | Active XDR-TB, incorrectly identified as DS-TB |
| T_xm_ | XDR-TB on inappropriate MDR-TB regimen |
| T_xs_ | XDR-TB on inappropriate DS-TB regimen |

**Table S2 Baseline parameter values**

| **Flow** | **Flow out of** | **Flow into** | **Baseline value** | **Rationale** |
| --- | --- | --- | --- | --- |
| Births | Enter model | S_A_ and S_B_ | 30 per 1000 population | Calibrated to a population growth rate of 1.5% per year.[^10^](#_ENREF_10) (Note that reported crude birth rate is similar, at 23.3 per 1000 population.) |
| Death rate | All S and L compartments | Exit model | 1 ÷ 68.24 | Reciprocal of United Nations reported life expectancy[^10^](#_ENREF_10) |
| Early progression | L_As_ | I_s_ | 0.092 over two years | Empiric reactivation data from the Netherlands[^11^](#_ENREF_11) |
|  | L_Am_  L_Ax_ | I_m_  I_x_ |  |  |
| Stabilisation | L_As_ | L_Bs_ | 0.908 over two years | Remainder of those not progressing early |
|  | L_Am_  L_Ax_ | L_Bm_  L_Bx_ |  |  |
| Late progression | L_Bs_ | I_s_ | 0.075 over twenty years | Approximate 5-10% lifetime risk[^12^](#_ENREF_12) |
|  | L_Bm_  L_Bx_ | I_m_  I_x_ |  |  |
| Detection | I_s_ | D_s_ | 90% ÷ 4.23/12 | 90% sensitivity of diagnostic algorithm (for all forms of TB), and  4.23 months median time to start treatment (pers comm MSF)  Note that this figure also leads to accurate replication of the reported incidence to prevalence ratio for 2015 |
|  | I_m_  I_x_ | D_mm_ & D_ms_  D_xx_ & D_xm_ & D_xs_ |  |  |
| Spontaneous recovery | I_s_ & D_ss_ | L_Bs_ | 30% of smear-positive and 80% of smear-negative patients recover over three years | Pre-chemotherapy era literature[^13^](#_ENREF_13) |
|  | I_m_, D_mm_ & D_ms_  I_x_, D_xx_, D_xm_  & D_xs_ | L_Bm_  L_Bx_ |  |  |
| Death untreated | I_s_ & D_ss_ | Exit model | 70% of smear-positive and 20% of smear-negative patients over three years | Pre-chemotherapy era literature[^13^](#_ENREF_13) |
|  | I_m_, D_mm_ & D_ms_  I_x_, D_xx_, D_xm_  & D_xs_ | Exit model  Exit model |  |  |
| Treatment commencement DS-TB | D_ss_ | T_Is_ | Over 1.142 weeks | Programmatic data (pers comm MSF) |
|  | D_ms_  D_xs_ | T_ms_  T_xs_ |  |  |
| Treatment commencement MDR-TB | D_mm_  D_xm_ | T_Im_  T_xm_ | Over 2.571 weeks or limited by treatment availability | Programmatic data (pers comm MSF) |
| Treatment commencement XDR-TB | D_xx_ | T_xx_ | Over 41 days or limited by treatment availability | Programmatic data (pers comm MSF) |
| Decrease in transmissibility DS-TB | T_Is_ | T_Ns_ | Over two weeks | Consistent with rapid decrease in infectiousness with treatment[^14^](#_ENREF_14)^,^[^15^](#_ENREF_15) |
| Regimen completion DS-TB | T_Ns_ | S_B_ | Over remaining 5 ½ months of regimen | WHO Guidelines[^16^](#_ENREF_16) |
| Interruption/failure proportion, DS-TB regimen | T_Is_ or T_Ns_ | I_s_ | 7.0% of total treatment rates | Programmatic data (pers comm MSF) |
| Death proportion, DS-TB regimen | T_Is_ or T_Ns_ | Exit model | 1.7% of total treatment rates | Programmatic data (pers comm MSF) |
| Decrease in transmissibility MDR-TB | T_Im_ | T_Nm_ | Over first three months of regimen duration | Approximately three months to decreased infectiousness[^14^](#_ENREF_14)^,^[^17^](#_ENREF_17) |
| Regimen completion MDR-TB | T_Nm_ | S_B_ | Until completion of the intended duration (average 24 or 10 months) | WHO Guidelines[^16^](#_ENREF_16) |
| Interruption/failure proportion, MDR-TB regimen (both WHO and short-course regimens) | T_Im_ or T_Nm_ | I_m_ | 26.4% of total treatment rates | Programmatic data (pers comm MSF) |
| Death proportion, MDR-TB regimen (both WHO and short-course regimens) | T_Im_ or T_Nm_ | Exit model | 8.7% of total treatment rates | Programmatic data (pers comm MSF) |
| Decrease in transmissibility XDR-TB | T_Ix_ | T_Nx_ | Over first eighth of regimen duration | Assumption from parameter for MDR-TB (under which infectiousness continues for the first one eighth of the treatment period) |
| Regimen completion XDR-TB | T_Nx_ | S_B_ | Until completion of the intended duration (average 24 months) | Programmatic data (pers comm MSF) |
| Interruption/failure proportion, XDR-TB regimen | T_Ix_ & T_Nx_ | I_x_ | 26.0 ÷ 77.4 (26.0% interrupt/fail out of the 77.4% for whom data are available) | Programmatic data (pers comm MSF) |
| Death proportion, XDR-TB regimen | T_Ix_ & T_Nx_ | Exit model | 28.7 ÷ 77.4 (28.7% death out of the 77.4% for whom data are available) | Programmatic data (pers comm MSF) |
| Treatment completion proportion, MDR-TB treated inappropriately with a DS-TB regimen | T_ms_ | S_B_ | 30% | Published literature (Karakalpakstan)[^18^](#_ENREF_18) |
| Treatment completion proportion, XDR-TB treated inappropriately with an MDR-TB regimen | T_xm_ | S_B_ | 20% | Estimate of outcomes for patients without access to new anti-TB drugs (pers comm MSF) |
| Treatment completion proportion, XDR-TB treated inappropriately with a DS-TB regimen | T_xs_ | S_B_ | 15% | Estimate |
|  |  |  | **Calculation** | |
| Force of infection DS-TB | S_A_ | L_As_ | Effective contact rate* × sum of compartments I_s_, D_ss_ and T_Is_ × proportion smear positive** ÷ population size | |
| Force of infection MDR-TB | S_A_ | L_Am_ | Effective contact rate* × sum of compartments I_m_, D_mm_, T_Im_ and D_ms_ × proportion smear positive** × relative fitness of MDR-TB† ÷ population size | |
| Force of infection XDR-TB | S_A_ | L_Ax_ | Effective contact rate* × sum of compartments I_x_, D_xx_, T_Ix_, D_xm_, T_xm_, D_xs_ and T_xs_ × proportion smear positive** × relative fitness of MDR-TB† ÷ population size | |
| Reduced force of infection DS-TB | S_B_, L_Bs_, L_Bm_  & L­_Bx_ | L_As_ | Force of infection DS-TB × 0.49[^19^](#_ENREF_19) | |
| Reduced force of infection MDR-TB | S_B_, L_Bs_, L_Bm_  & L_Bx_ | L_Am_ | Force of infection MDR-TB × 0.49 | |
| Reduced force of infection MDR-TB | S_B_, L_Bs_, L_Bm_  & L_Bx_ | L_Ax_ | Force of infection XDR-TB × 0.49 | |
| **Proportion** | **Numerator** | **Denominator** | **Baseline value** | **Rationale** |
| Infectious proportion | 4030 (SS+)  + 0.24 × 6137 (SS-) | 4030 (SS+) + 6137 (SS-) + 3965 (extrapul) | 38.9% | 2012 Global TB Report numbers[^20^](#_ENREF_20) and estimate of 0.24 relative infectiousness of smear-negative pulmonary cases[^21^](#_ENREF_21) |
| Relative infectiousness | Infectiousness of MDR-TB or XDR-TB on inappropriate regimen | Infectiousness of untreated MDR-TB | 75% | Assumption |
| MDR-TB identification | I_m_ to D_mm_  *or*  I_x_ to I_xx_ & I_xm_ | I_m_ to D_mm_ & D_ms_  *or*  I_x_ to I_xx_, I_xm_, I_xs_ | 69.1% | Programmatic data (pers comm MSF) |
| XDR-TB identification | I_x_ to I_xx_ | I_x_ to I_xx_, I_xm_ | 60% | Estimate (pers comm MSF)  Proportion of XDR-TB patients correctly identified as such, among XDR-TB patients correctly identified as MDR-TB |
| BCG vaccination | Enter S_B_ | Enter S_A_ & S_B_ | 99% coverage | UNICEF data[^22^](#_ENREF_22) |
| Amplification to MDR-TB with interruption/failure from DS-TB treatment | T_Is_ & T_Ns_ to  I_m_ | T_Is_ & T_Ns_ to I_s_ & I_m_ | 2/15 | Proportion of interrupting/failing patients amplifying to MDR-TB (N.B. amplification does not occur following successful treatment)[^17^](#_ENREF_17) |
| Amplification to XDR-TB with interruption/failure from MDR-TB treatment | T_Im_ & T_Nm_  to I_x_ | T_Im_, T_Nm_ & T_ms_  to I_x_ | 2/15 | Assumed to be equal to the proportion for amplification from DS-TB to MDR-TB, given that each of these steps require resistance to two additional antibiotics to which the strain was previously susceptible |

*Effective contact rate calibrated to target incidence. **Proportion smear positive derived from Global TB Report 2013.[^23^](#_ENREF_23) †Value of 0.6 is calibrated to model dynamics and based on consensus that fitness cost is likely to be moderate at most.[^8^](#_ENREF_8) ‡Evidence for low, but non-zero treatment success rates in patients treated with inappropriate regimens includes ^[24](#_ENREF_24)^. Extrapul, extrapulmonary; MSF, Médecins sans Frontières; SS+, smear-positive; SS-, smear-negative; UNICEF, United Nations International Children’s Emergency Fund; WHO, World Health Organization.

**
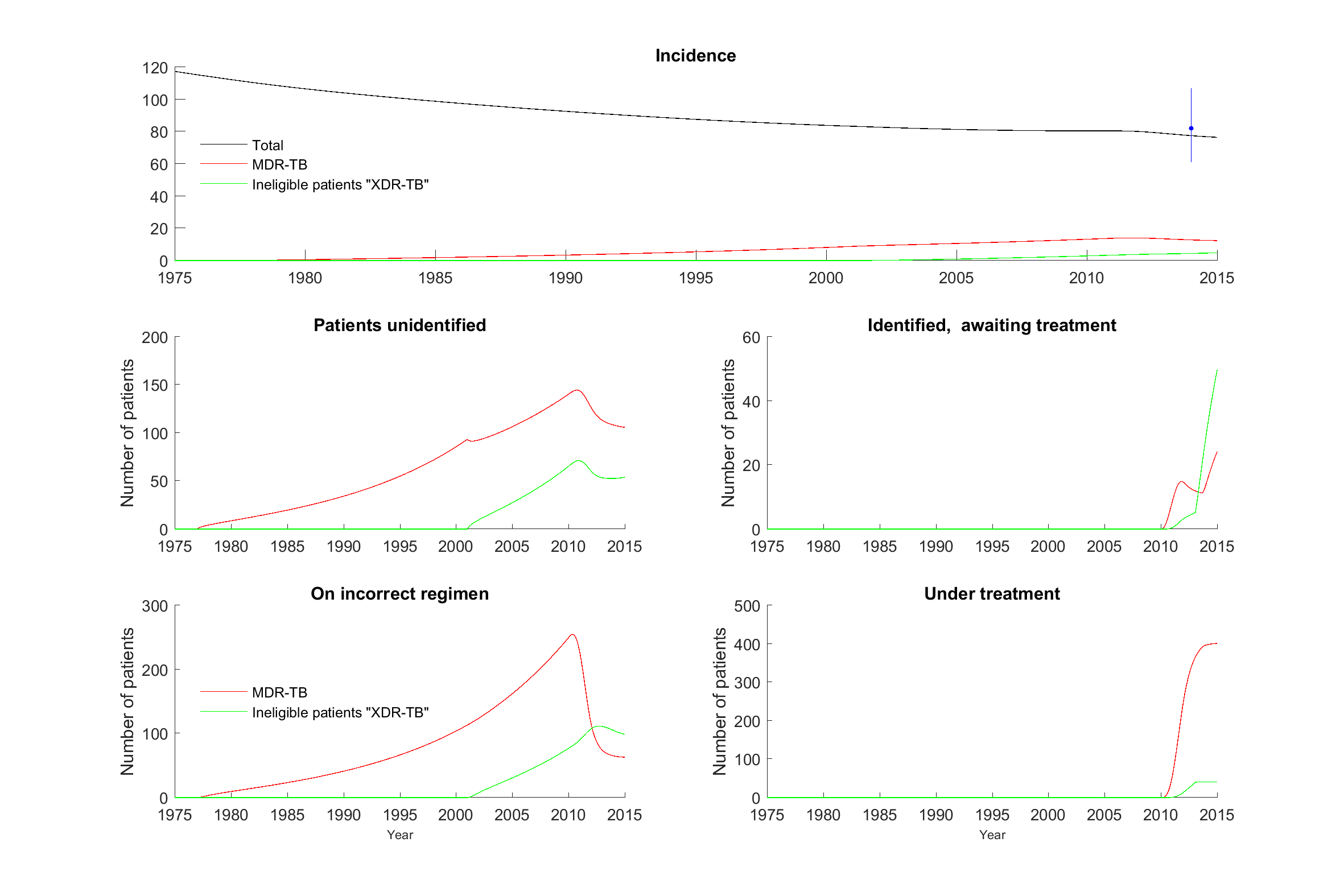
**

**Figure S1 Calibration**

Upper panel shows the overall dynamics of total incidence and MDR-TB over the treatment scale-up and MDR-TB emergence period. Incidence to which model was calibrated is marked with blue circle for point estimate and vertical blue bar for range. Lower four panels show a similar time period and the relative distribution of patients with MDR-TB (red) and XDR-TB (green) over this period in relation to their programmatic status. Note that as no MDR-TB identification was available prior to 2010, all patients were considered to have been either on no treatment or on an inappropriate first line regimen prior to this time.


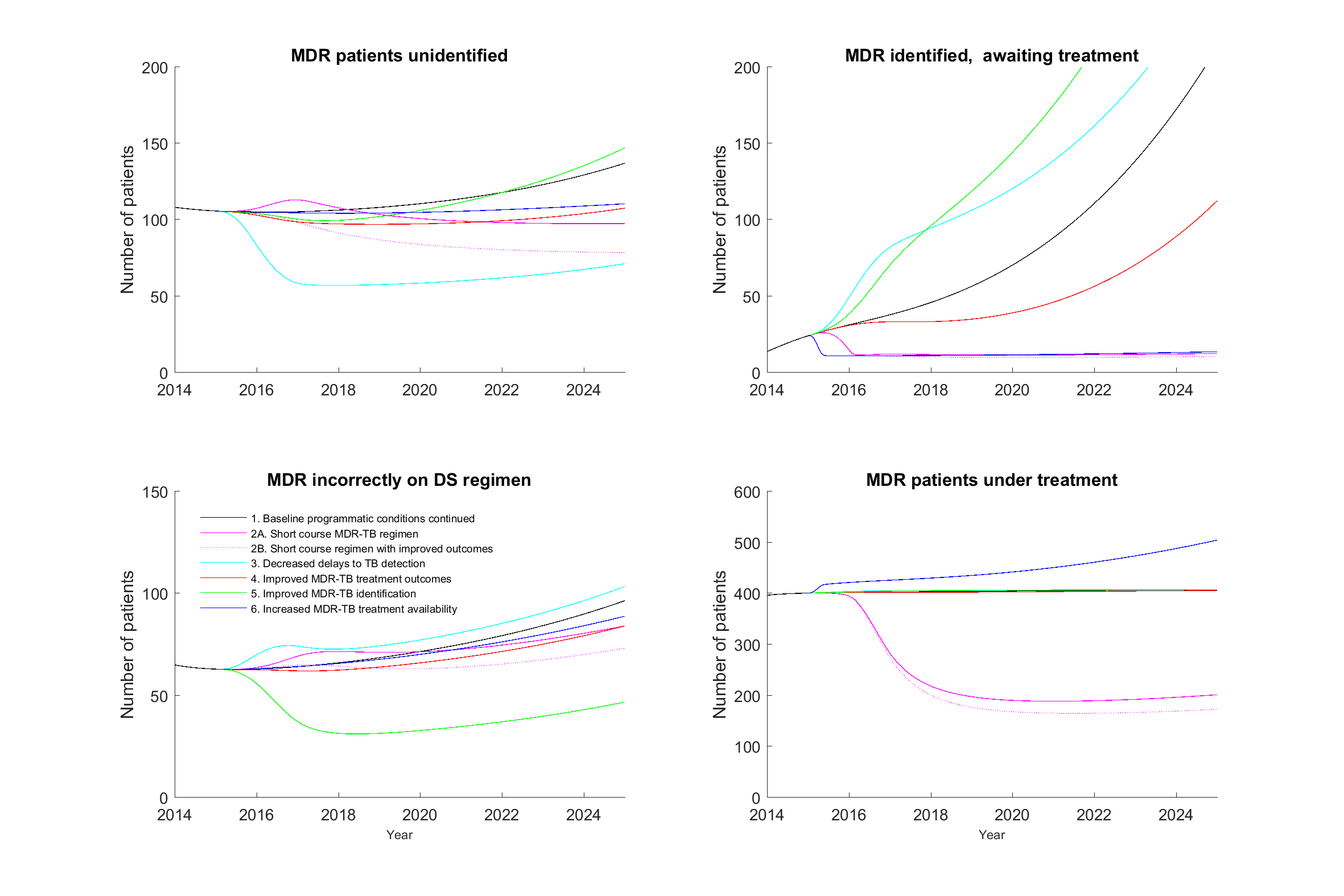


**Figure S2 Population distribution by scenario**

Note that scenario colours are the same as for Figure 3 (main text).


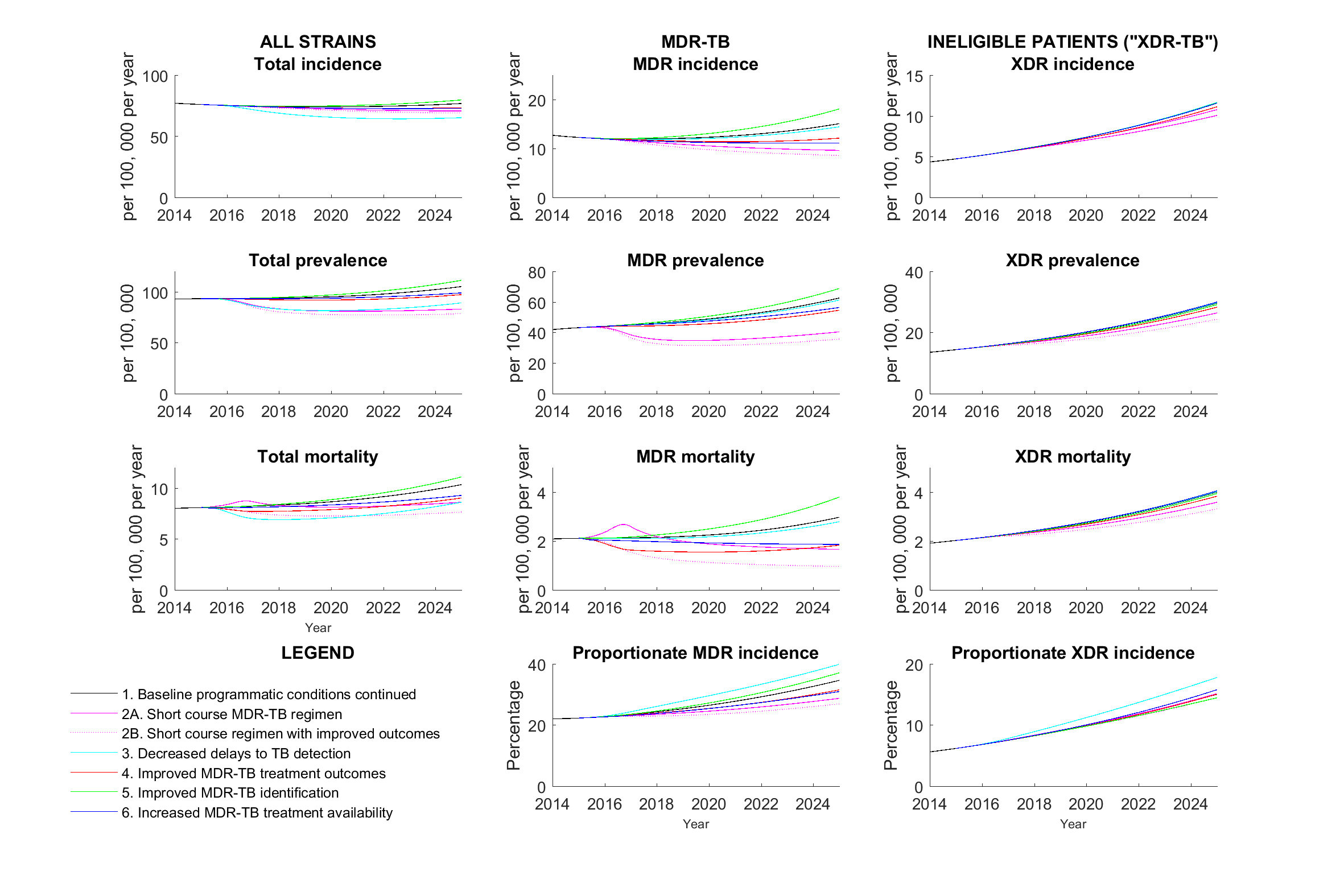


**Figure S3 Scenario results under the alternative MDR-TB burden assumption**

Strains are presented by columns of panels, while disease burden outcomes are presented by row. Legend for all plots is presented in the lower left panel.


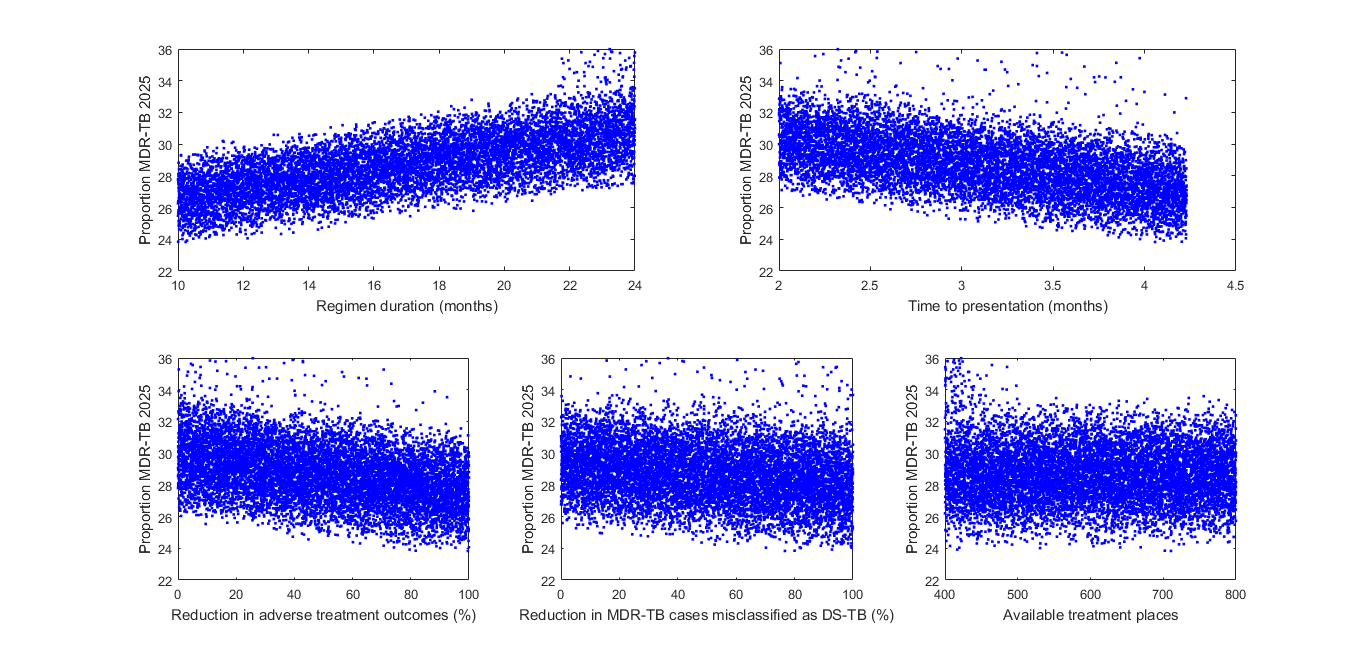


**Figure S4 Sensitivity analysis on outcome of proportionate incidence of MDR-TB 2025**


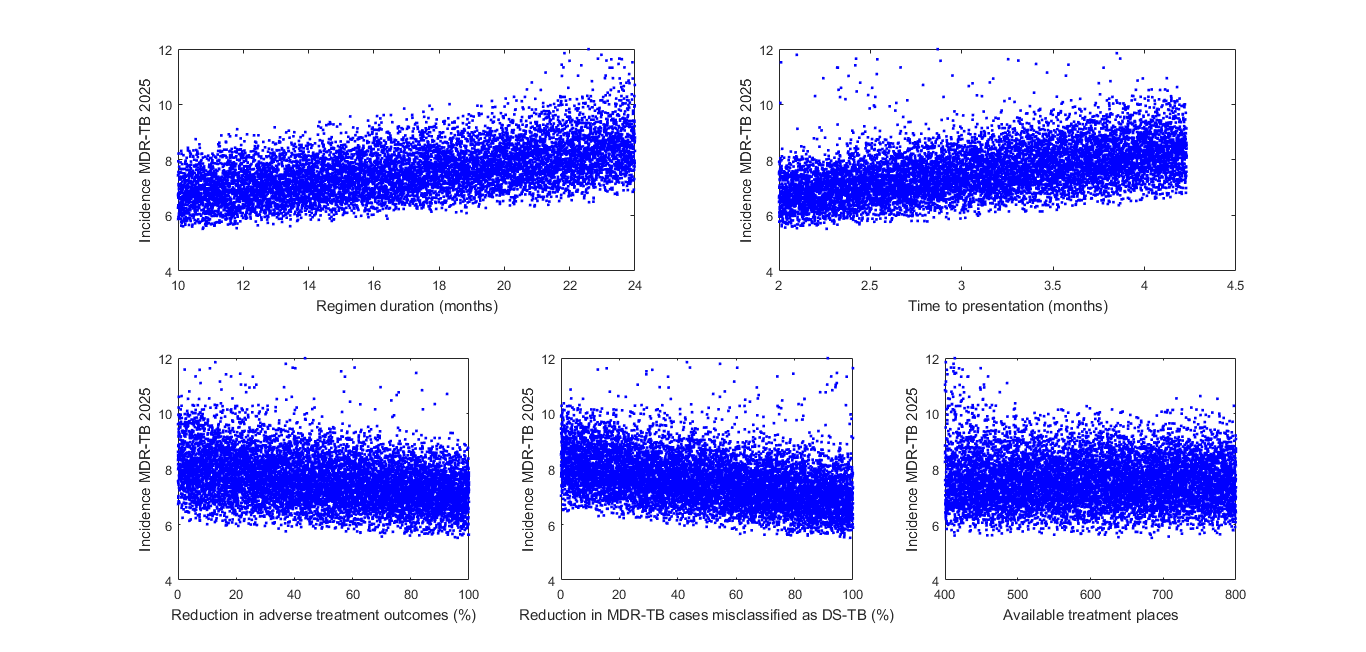


**Figure S5 Sensitivity analysis on outcome of absolute incidence of MDR-TB 2025**

1 Trauer JM, Denholm JT, McBryde ES. Construction of a Mathematical Model for Tuberculosis Transmission in Highly Endemic Regions of the Asia-Pacific. *J Theor Biol* 2014; 358: 74-84.

2 Trauer JM, Denholm JT, Waseem S, Ragonnet R, McBryde ES. Scenario Analysis for Programmatic Tuberculosis Control in Western Province, Papua New Guinea. *Am J Epidemiol* 2015: In press.

3 Ragonnet R, Trauer JM, Denholm JT, Geard NL, Hellard M, McBryde ES. Vaccination Programs for Endemic Infections: Modelling Real versus Apparent Impacts of Vaccine and Infection Characteristics. *Sci Rep* 2015; 5: 15468.

4 World Health Organization. Companion Handbook to the WHO guidelines for the programmatic management of drug-resistant tuberculosis. Geneva, 2014.

5 Ulmasova DJ, Uzakova G, Tillyashayhov MN, et al. Multidrug-resistant tuberculosis in Uzbekistan: results of a nationwide survey, 2010 to 2011. *Euro Surveill* 2013; 18.

6 Boehme CC, Nabeta P, Hillemann D, et al. Rapid molecular detection of tuberculosis and rifampin resistance. *N Engl J Med* 2010; 363: 1005-15.

7 World Health Organization. Uzbekistan, Tuberculosis Profile. 2015. <https://extranet.who.int/sree/Reports?op=Replet&name=%2FWHO_HQ_Reports%2FG2%2FPROD%2FEXT%2FTBCountryProfile&ISO2=UZ&LAN=EN&outtype=html> Date last accessed: 18th January 2016.

8 Cohen T, Sommers B, Murray M. The effect of drug resistance on the fitness of Mycobacterium tuberculosis. *Lancet Infect Dis* 2003; 3: 13-21.

9 Van Deun A, Maug AK, Salim MA, et al. Short, highly effective, and inexpensive standardized treatment of multidrug-resistant tuberculosis. *Am J Respir Crit Care Med* 2010; 182: 684-92.

10 United Nations Department of Economic and Social Affairs PD. World Population Prospects, the 2015 Revision. 2010-2015. <http://esa.un.org/unpd/wpp/DVD/> Date last accessed: 17th August 2015.

11 Sloot R, Schim van der Loeff MF, Kouw PM, Borgdorff MW. Risk of Tuberculosis after Recent Exposure. A 10-Year Follow-up Study of Contacts in Amsterdam. *Am J Respir Crit Care Med* 2014; 190: 1044-52.

12 Blower SM, McLean AR, Porco TC, et al. The intrinsic transmission dynamics of tuberculosis epidemics. *Nat Med* 1995; 1: 815-21.

13 Tiemersma EW, van der Werf MJ, Borgdorff MW, Williams BG, Nagelkerke NJ. Natural history of tuberculosis: duration and fatality of untreated pulmonary tuberculosis in HIV negative patients: a systematic review. *PLoS One* 2011; 6: e17601.

14 Dharmadhikari AS, Mphahlele M, Venter K, et al. Rapid impact of effective treatment on transmission of multidrug-resistant tuberculosis. *Int J Tuberc Lung Dis* 2014; 18: 1019-25.

15 Riley RL, Mills CC, O'Grady F, Sultan LU, Wittstadt F, Shivpuri DN. Infectiousness of air from a tuberculosis ward. Ultraviolet irradiation of infected air: comparative infectiousness of different patients. *Am Rev Respir Dis* 1962; 85: 511-25.

16 World Health Organization. Treatment of Tuberculosis Guidelines. Geneva, Switzerland: WHO, 2010.

17 Kurbatova EV, Gammino VM, Bayona J, et al. Predictors of sputum culture conversion among patients treated for multidrug-resistant tuberculosis. *Int J Tuberc Lung Dis* 2012; 16: 1335-43.

18 Cox H, Kebede Y, Allamuratova S, et al. Tuberculosis recurrence and mortality after successful treatment: impact of drug resistance. *PLoS Med* 2006; 3: e384.

19 Colditz GA, Brewer TF, Berkey CS, et al. Efficacy of BCG vaccine in the prevention of tuberculosis. Meta-analysis of the published literature. *JAMA* 1994; 271: 698-702.

20 World Health Organization. Global Tuberculosis Report 2015. 2015. <http://apps.who.int/iris/bitstream/10665/191102/1/9789241565059_eng.pdf?ua=1> Date last accessed: 12th November 2015.

21 Tostmann A, Kik SV, Kalisvaart NA, et al. Tuberculosis transmission by patients with smear-negative pulmonary tuberculosis in a large cohort in the Netherlands. *Clin Infect Dis* 2008; 47: 1135-42.

22 UNICEF. Uzbekistan, Statistics. 2012. <http://www.unicef.org/infobycountry/uzbekistan.html> Date last accessed: 14th March 2014.

23 World Health Organization. Global Tuberculosis Report 2013. 2013. <http://apps.who.int/iris/bitstream/10665/91355/1/9789241564656_eng.pdf2011>.

24 Espinal MA, Kim SJ, Suarez PG, et al. Standard short-course chemotherapy for drug-resistant tuberculosis: treatment outcomes in 6 countries. *JAMA* 2000; 283: 2537-45.
